# Economic stress or random variation? Revisiting German reunification as a natural experiment to investigate the effect of economic contraction on sex ratios at birth

**DOI:** 10.1186/1476-069X-13-117

**Published:** 2014-12-22

**Authors:** Sebastian Schnettler, Sebastian Klüsener

**Affiliations:** Department of Sociology, University of Konstanz, Box 40, Universitätsstr. 10, 78457 Konstanz, Germany; Max Planck Institute for Demographic Research, Konrad-Zuse-Str. 1, 18057 Rostock, Germany

**Keywords:** Sex ratio at birth, Economic decline, Unemployment, Economic stress, German reunification, Natural experiment

## Abstract

**Background:**

The economic stress hypothesis (ESH) predicts decreases in the sex ratio at birth (SRB) following economic decline. However, as many factors influence the SRB, this hypothesis is difficult to test empirically. Thus, researchers make use of quasi-experiments such as German reunification: The economy in East, but not in West Germany, underwent a rapid decline in 1991. A co-occurrence of a decline in the East German SRB in 1991 has been interpreted by some as support for the ESH. However, another explanation might be that the low SRB in 1991 stems from increased random variation in the East German SRB due to a drastically reduced number of births during the crisis. We look into this alternative random variation hypothesis (RVH) by re-examining the German case with more detailed data.

**Methods:**

Our analysis has two parts. First, using aggregate-level birth register data for all births in the period between 1946 and 2011, we plot the quantum and variance of the SRB and the number of births and unemployment rates, separately for East and West Germany, and conduct a time series analysis on the East German SRB over time. Second, we model the odds for a male birth at the individual level in a multiple logistic regression (1991–2010, ~13.9 million births). Explanatory variables are related to the level of the individual birth, the mother of the child born, and the regional economic context.

**Results:**

The aggregate-level analysis reveals a higher degree of variation of the SRB in East Germany. Deviations from the time trend occur in several years, seemingly unrelated to economic development, and the deviation in 1991 is not statistically significant. The individual-level analysis confirms that the 1991-drop in the East German SRB cannot directly be attributed to economic development and that there is no statistically significant effect of economic development on sex determination in East or West Germany.

**Conclusion:**

Outcomes support the RVH but not the ESH. Furthermore, our results speak against a statistically significant effect of the reunification event itself on the East German SRB. We discuss the relative importance of behavioral and physiological responses to macro-level stressors, a distinction that may help integrate previously mixed findings.

## Background

For human populations, a sex ratio at birth (SRB) of about 105 boys to 100 girls is seen as "natural" [1]. But a number of studies have found that the SRB in a population may be affected by individual-level stressors and macro-social shocks that lead to short-term deviations from the time trend [2, 3]. This renders sex ratio biasing a topic of interest for both the biological and the social sciences. Empirical findings on the direction of the effect of stress on the SRB have been equivocal: On the one hand, some individual-level stressors have been found to be linked to SRB decreases. Examples of these kinds of stressors are occupational and psychological stress [4, 5]. In addition, a range of macro-social shocks, like short wars [6–9], terrorist attacks [10, 11], and natural disasters [12, 13] have also been linked to SRB decreases. On the other hand, SRB increases have occurred, for example, in the belligerent countries after the First and Second World Wars [14–16].

Here we are specifically concerned with the effects of economic contraction on the SRB. Also with regard to economic contraction, empirical results are mixed: Catalano and Bruckner [17], for instance, reported a negative association between economic development, measured as the percentage change in private consumption, and the SRB in Sweden. Helle et al. [15] likewise found a negative, yet smaller, association between GDP and the SRB in Finland between 1865 and 2003, controlling for a range of additional contextual influences on the SRB. However, a time series analysis examining the links between consumption data and the SRB for Poland between 1956 and 2005, Żądzińska et al. [18], found no association. A positive association between economic conditions and the SRB appeared in Cuba after the economic collapse in the early 1990s [19]. But this was later attributed to a temporary, yet systematic bias in official birth registration [20, 21], see also [22], and [23] for earlier speculation on a different mechanism.

The mixed empirical pattern has led researchers to ponder the potential mechanisms at play in linking economic development to the SRB. Recently, Grant and collaborators proposed a model that integrates divergent explanations into a combined framework and may help to explain why the SRB tends to increase after some types of crises, but to decrease after other ones [24–26]. The focus in this framework is on two mechanisms that may be part of an adaptive system of SRB variation. First, it is well established that male fetuses are more vulnerable to stressors than female fetuses at any developmental stage [27, 28]. Thus, sex-differential mortality in utero in response to stress can explain SRB decreases. Second, concentrations of follicular testosterone and glucose, which are associated with a higher probability of conceiving a boy, tend to increase with stress, and can therefore lead to an increase of the SRB [24, 25, 29]. Direct empirical evidence for the association between both follicular testosterone [30–32] and glucose levels and the sex of subsequent embryos [33] has been obtained from experimental animal studies. Based on these findings, it appears that in mammals the uterine environment influences the ovum prior to conception, making the ovum more or less likely to accept a spermatozoon carrying a Y over an X chromosome [25]. This mechanism could be seen as an evolutionary response to male fragility, stabilizing the SRB in a population in the medium term when stressful conditions persist [24].

The timing of stress exposure at conception and during pregnancy plays an important role in this framework, and allows us to make rough predictions about the direction of the expected SRB bias in a population. If stress occurs around the time of conception, more males will be conceived; but if stress persists during pregnancy, more males will die in utero, and the SRB might be normal. If stress occurs around the time of conception but subsides during pregnancy, more males will be conceived and survive pregnancy, resulting in a male-biased SRB. Finally, under normal conditions, there will be no overproduction of male fetuses. If stress occurs during pregnancy and more males die in utero, the SRB in a population may decrease relative to normal conditions. These two mechanisms, pre-conception testosterone and glucose levels and in-utero male vulnerability, together explain some of the inconsistencies in the results of previous studies [25]. Studies that used a more fine-grained timing of measurement of stress in relation to the timing of conception showed that effects on the SRB, which were previously thought to be extremely small and hard to detect [34], may in fact be considerably larger than commonly believed [35–37].

In addition to the challenge of measurement timing, researchers are faced with the problem of confounding by additional influences on the SRB. A large number of potential correlates with the SRB have been suggested, including birth order, parental age, coital frequency, parental hormone levels, genetic effects, birth registration changes, and exposure to certain toxins [16, 33, 38, 40]. For this reason, Zorn et al. [41] expressed skepticism about the usefulness of correlational tests: "Because of the interaction of multiple factors, the aetiology of sex ratio changes after stressful events is not expected to be elucidated in the very near future. Today we are not in the position to make determinate conclusions regarding the association between stress and sex ratio changes, as the existing studies on the relationship between testosterone and adverse events lack appropriate methodologies. These studies are not controlled, much less are they randomized." Also a recent review on the influence of economic contraction and birth outcomes concludes that the association between economic stress and the SRB remains speculative [42].

Against this background, the case of German reunification becomes interesting as a natural experiment to test the influence of economic stress on the SRB: In this case, we have two very similar populations with a common institutional framework following reunification. Yet, post-reunification economic decline selectively affected East Germany in 1991 and in subsequent years [43]. Making use of this case, Catalano [44] showed on the basis of East and West German aggregate data for the period between 1945 and 1999 that the SRB deviated significantly downward from the time trend in East Germany in 1991. This result is consistent with previous findings of a negative association between the SRB and economic conditions, and may be attributable to rapid economic decline in East Germany around 1991.

Our study is motivated by the recent emphasis on statistical challenges in research on the SRB [34, 45]. Given the high relevance that is attributed to German reunification to test the economic stress hypothesis (ESH), we will re-evaluate this case using richer data and more refined analytical methods that address these statistical challenges. We will put particular emphasis on aspects of statistical power and randomness. As expected effect sizes in research on the SRB are often small, they require very large samples to detect these small effects. Whereas generally the kind of data we have available here do provide a considerable degree of statistical power, it should be noted that the underlying statistical power differs between East and West Germany. The number of births in East Germany from which the SRB data are generated is lower. This is due to the smaller size of the population in East Germany compared to that in West Germany. Of particular concern for our analysis is that in East Germany the number of births even decreased further to very low levels after 1990, a change that itself has been attributed to the economic transition crisis after 1990 [46, 47]. The latter might have increased volatility in the annual East German SRB after 1990, which renders the identification of outliers from the time trend particularly difficult [48, 49]. Comparable to the multiple comparison problem in other types of analyses, a conventional level of statistical significance of 5% would lead to the detection of SRB outliers as false positives in about five years over a period of 100 years, or in about three years over the 65 year period between 1946 and 2011 observed in our study (.05*65 = 3.25) [34]. The considerations outlined in this paragraph motivated us to evaluate higher random volatility in the East German SRB due to the rapid post-unification fertility decline as an alternative explanation for the low East German SRB in 1991. To this hypothesis, which we propose as an alternative to the ESH, we will refer to as "random variation hypothesis" (RVH).

To determine if the deviation from the time trend in 1991 is a real outlier and not just a random deviation from the time trend requires careful statistical modeling that takes the different degree of volatility in the SRB over time and between the two parts of Germany into account. Therefore, we revisit the case of German reunification by methods allowing to control for these aspects and analyzing a longer time series for East and West Germany up to and including the year 2011 and by drawing on individual-level data on births that occurred in East and West Germany between 1991 and 2010. This allows us to test the ESH against the RVH: First, and in line with the argument made by Catalano [44], the SRB deviation from the time trend in 1991 may be related to differences in the economic development of post-reunification East Germany relative to that of West Germany. Second, and according to the RVH, the deviations in 1991 could reflect higher random variation due to a small number of births in East Germany after reunification. We test both hypotheses using time-sensitive analyses that take into account Grant’s model of divergent mechanisms outlined above.

## Methods

### Aggregate-level analysis

Our aggregate-level analysis is based on yearly SRB data for Germany covering the period between 1946 and 2011. This data we obtained from the Human Mortality Database [50]. Our analytical strategy for this part of the analysis builds on and extends the approach used by Catalano in his original test of the ESH [44] with the difference that in our regression models we used the percentage of male births instead of the sex ratio per se as the dependent variable. This is recommended in the literature to avoid common errors in interpretation of the results [51]. Specifically, we proceeded by conducting a time-series analysis of the percentage of male births in East Germany to account for temporal autocorrelation present in the time series. Furthermore, in all models we controlled for the percentage of male births in West Germany during the same years to account for unobserved heterogeneity shared by East and West Germany. To test if the percentage of male births in 1991 deviates significantly from the time trend, all models also include a dummy variable for 1991. We extended Catalano’s approach by accounting for the considerable degree of heteroskedasticity present in the time series data.

Specifically, we compare five models in the Results section, which are described in detail in the statistical appendix: Model 1 is a simple linear regression model in which time is entered as a continuous covariate. Model 2 accounts for temporal autocorrelation with an autoregression component (AR1) and additionally includes a moving average (MA1). The "1" indicates that we are working with lags of one year. We chose the combination of AR1 and MA1 as it provided the best goodness of fit as compared to a range of alternative AR and MA specifications. Whereas Model 2 adjusts for temporal autocorrelation, it doesn’t correct for the considerable degree of heteroskedasticity present in the data. In Models 3–5 we therefore accounted for heteroskedasticity by implementing different weighting procedures in weighted least squares regressions. In Model 3, weights are based on standard errors obtained from an autoregressive conditional heteroskedasticity (ARCH) model [52]. Since this left a considerable amount of heteroskedasticity, we tested additional weighted least squares specifications. Given that the number of births in East Germany dropped considerably after reunification, in Model 4 the weights were obtained from the underlying sample sizes, that is, the total number of births that occurred in East and West Germany in each year. In Model 5, we combined the two types of weights from Models 3 and 4 multiplicatively [53].

### Individual-level analysis

According to the theory by Valerie Grant, outlined above, the different timing of stress exposure may lead to diametrical effects on sex determination that could neutralize each other in annual aggregate data. We therefore amended the aggregate-level analysis with an individual-level analysis that allowed a more time-sensitive examination of the potential effect of economic contraction on sex determination. This was achieved by using individual-level data from the German birth register that includes information on the month of birth. To this data we obtained access through the Data Research Center of the German statistical offices [54]. 100%-samples were available for almost all federal states for the years 1991 to 2010^a^. We excluded multiple births and births that occurred in Berlin, as the data did not allow us to distinguish between former East and West Berlin. This left a total of close to 13.9 million births which we considered in our analysis. For these, in addition to information on the month of birth, we also had a number of individual-level attributes available. Furthermore, we could link the birth data to unemployment data at the level of the federal states. The added variation of unemployment rates through the monthly and more fine-grained regional specification allowed to control for spatial and temporal variation in the magnitude of the economic transition crises in East Germany following reunification. Our analytical strategy in this part of the analysis was to model sex determination at birth as a binary random process and to use logistic regression to analyze how offspring sex is associated with changes in unemployment rates [2].

Consistent with previous research from animal experiments, which showed that the effect of changes in conditions are more influential on the SRB than absolute resource levels [33], we operationalized economic stress as the change in the regional unemployment rate in percentage points across a three-month period, timed relative to the month of the individual birth (t_0_). And in response to the theoretical arguments outlined above about expected differences in the strength and direction of stress effects at different times between conception and pregnancy, we first conducted a sensitivity analysis to determine at which time and in which direction unemployment change affects the sex of the respective child most strongly. To this purpose we used different time lags of unemployment change, measured at the level of 15 of the 16 federal states of Germany (excluding Berlin)^b^. For example, a lag of four would indicate unemployment change between the seventh (t_-7_) and fourth month (t_-4_) prior to the respective birth (t_0_). And a lag of eight would measure unemployment change between the eleventh and eighth month prior to a birth (t_-11_:t_-8_).

Given the large amount of cases in our sample and limitations in computing power, we conducted the sensitivity analysis on appropriate unemployment change lags on the full sample but without any additional covariates. Based on the results of the sensitivity analysis, we proceeded in our analysis with the time lag of unemployment change that showed the highest association with the odds for a male birth (lag 4). In a series of multiple logistic regression models, we tested whether unemployment change and the occurrence of a birth in East Germany in 1991 showed a statistically significant and robust effect on the odds for a male birth. In a first step, we extended the bivariate version of the model to include a dummy variable for the year in which a respective birth occurred (1991 vs. any other year), a dummy for the region of birth (West or East Germany), and an interaction between the two. This allowed us to check whether the SRB in 1991 deviated from the trend in East but not in West Germany. In a second step, we added additional control variables on the level of the individual birth: maternal characteristics at the time of birth (employment status: employed or not employed, age, marital status: married or unmarried, and nationality: German or foreign), and month of birth to adjust for seasonality effects [1, 55]. Although the age of both parents at the time of birth has been linked to sex composition [56], we could not control for paternal age, as this information was not available for all births^c^. Our data were also limited with regard to parental socioeconomic status. We therefore could not control for a possible Trivers-Willard effect, which would predict a biased probability of having a male or a female birth depending on socioeconomic status [37, 57–59]. In a third step, we added dummy variables for the federal state in which the birth took place to account for possible unobserved heterogeneity at the level of these states.

In the course of the modeling process, we also tested interaction effects. In one case, this served to test if unemployment change had a stronger effect in 1991, that is, at the beginning of a multi-year increase in unemployment in East Germany after reunification (acute stress), than in any other year (chronic stress). And, in another case, this served to test if the effect of unemployment change on sex determination is moderated by mother’s employment status.

## Results

### Aggregate-level data analysis (1946–2011)

**Figure 1:**
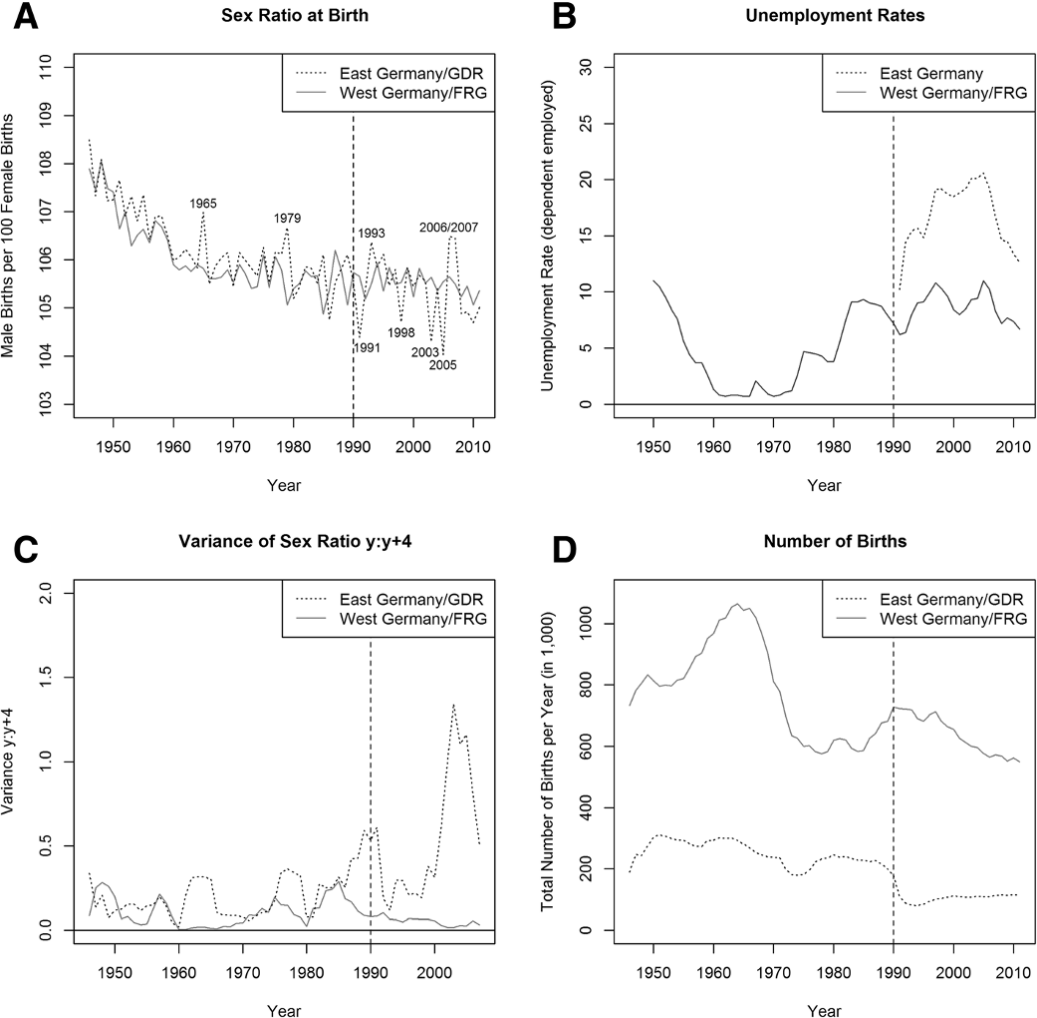
**SRB, unemployment rate, moving SRB variance, and number of births (by region and year, 1946–2011).** In Panel B (unemployment rates) data limitations do not allow us to distinguish between East and West Berlin after 2000. Thus, from 2001 onward, we excluded Berlin from our trend data. Data sources: Human Mortality Database [50] and Federal Employment Agency, own calculations.

Visual inspection of the SRB in East and West Germany over time (see Panel A, Figure 1) shows for East Germany multiple deviations from the time trend - some of which are about as large as the 1991 deviation pointed out by Catalano [44] and some of which are smaller. In three years, the larger of these deviations were in the direction of SRB increases (1965, 1979, and 2006/2007), while in three other years the deviations were in the direction of SRB decreases (1998, 2003, 2005). Unemployment rates in East Germany increased sharply in 1991 and 1992, and continued to rise in subsequent years, with two exceptions: 1995–1996 and 1999–2000. In 2006, unemployment peaked and then declined (see Panel B, Figure 1).

The trend data also show the smaller and decreasing number of births in East Germany as compared to West Germany which motivated us to propose and test the RVH (see Panel D, Figure 1). Throughout the period studied, the SRB seems to fluctuate more in East than in West Germany (see Figure 1, Panel A). A moving variance, calculated over five-year intervals, indicates that the variance of the SRB in East Germany is higher, and rises with falling birth rates after reunification^d^ (see Figure 1, Panel C). The results of the regular linear regression on the percentage of male births in East Germany show a significant deviation for the 1991 dummy (see Table 1, Model 1). Yet this effect ceases to be statistically significant once we account for temporal autocorrelation (Model 2). Among Models 3–5, which account for heteroskedasticity, Model 4 has the best fit with the lowest AIC value of the three models. However, Model 2 provides the best fit of all models with the lowest AIC value across all five models. Overall, we see that the effect of 1991 is not robust to different model specifications. The two models with the best fit to the East German time series of the percentages of male births, Models 2 and 4, suggest that the effect of a birth taking place in 1991 does not amount to a statistically significant deviation from the time trend.

**Table 1:** **Time series regression on the percentage of male births in East Germany, 1946-2011**

	Model 1	Model 2	Model 3	Model 4	Model 5
	Least squares model	ARMA (1,1)	Weighted least squares models		
			ARMA(1,1), ARCH(2)	Weighted by birth counts	Weights of models 3 & 4 combined
	β ***(SE)***	β ***(SE)***	β ***(SE)***	β ***(SE)***	β ( ***SE)***
(Intercept)	30.225***	20.833***	30.282***	30.918***	31.221***
	*(8.729)*	*(.731)*	*(8.220)*	*(8.069)*	*(7.838)*
Time Series	.581***	.724***	.580***	.575***	.569***
(West Germany)	*(.136)*	*(.014)*	*(.127)*	*(.122)*	*(.117)*
Year	-.004***	-.003***	-.004***	-.005***	-.005***
	*(.001)*	*(.000)*	*(.001)*	*(.001)*	*(.001)*
Year = 1991	-.300*	-.138	-.299*	-.294	-.293
	*(.120)*	*(.108)*	*(.134)*	*(.152)*	*(.174)*
AR(1)		.549***	.145		
		*(.125)*	*(.385)*		
MA(1)		-1.000***	-.486		
		*(.044)*	(.365)		
AIC	-88.2	-95.1	-91.5	-92.8	-92.7
R^2^	.683	-	.698	.710	.716
Adj. R^2^	.668	-	.684	.696	.702
Num. obs.	66	66	66	66	66

Data source: Human Mortality Database [50], own calculations; ***p < .001, **p < .01, *p < .05.

### Analysis of individual-level data from the German birth register (1991–2010)

In our individual-level data analysis, we first turn to outcomes of a series of bivariate logistic regression models. These are part of our sensitivity analysis of the strength and direction of the effects of various time lags of our unemployment change indicator on the odds of a male over a female birth (see Table 2). The results show that unemployment change seems to have a significantly positive effect on the odds of a male birth at lags three to six. At lag four, that is, for unemployment change between the seventh and the fourth month prior to birth, which roughly corresponds to the second trimester of an average pregnancy, the effect is largest (OR: 1.003; SE: .001).

**Table 2:** **Bivariate logistic regressions of male birth, controlling for unemployment change**

Lags of 3-month unemployment change, relative to month of birth (t)	Odds ratio ***(SE)***	Constant ***(SE)***
Lag 1 (t_-4_:t_-1_)	.9990 *(.0006)*	1.0657*** *(.0006)*
Lag 2 (t_-5_:t_-2_)	.9998 *(.0005)*	1.0656*** *(.0006)*
Lag 3 (t_-6_:t_-3_)	1.0014** *(.0005)*	1.0656*** *(.0006)*
Lag 4 (t_-7_:t_-4_)	1.0028*** *(.0005)*	1.0656*** *(.0006)*
Lag 5 (t_-8_:t_-5_)	1.0025*** *(.0005)*	1.0655*** *(.0006)*
Lag 6 (t_-9_:t_-6_)	1.0012* *(.0005)*	1.0656*** *(.0006)*
Lag 7 (t_-10_:t_-7_)	.9997 *(.0005)*	1.0657*** *(.0006)*
Lag 8 (t_-11_:t_-8_)	.9995 *(.0005)*	1.0657*** *(.0006)*
Lag 9 (t_-12_:t_-9_)	1.0004 *(.0006)*	1.0656*** *(.0006)*
N (same for each model)	13 863 433	
AIC (same for each model)	19 200 000	

Data sources: German Birth Register 1991–2010 [54] and Federal Employment Agency; East and West Germany (Berlin excluded), own calculations; unemployment data for 1990 partly based on secondary sources and imputations; ***p < .001, **p < .01, *p < .05.

Next, we continued with a series of multiple logistic regression models in which we tested the association of unemployment change at lag four with sex determination (see Table 3). In Model 1, we added dummy variables for year of birth (1991 vs. any other year), region of birth (East vs. West Germany), and the interaction between the two. The coefficients for the main effect of year of birth (OR: 1.008, SE: .003), region of birth (OR: .996, SE: .002), and the interaction (OR: .985, SE: .007) are all statistically significant. With a marginal odds ratio of .996, the odds for a male birth in East Germany in years other than 1991 were thus higher than the marginal odds for a male birth in East Germany in 1991 (OR=.989=1.008*.996*.985). After controlling for these two main effects and the interaction effect, the effect of unemployment change at lag four remains the same as in the bivariate model (OR: 1.003; SE: .001).

**Table 3:** **Multiple logistic regression of male birth**

	Model 1		Model 2		Model 3	
	OR	(SE)	OR	(SE)	OR	(SE)
(Constant)	1.066***	(.001)	1.070***	(.003)	1.072***	(.003)
Unemployment change, lag 4 (t_-7_:t_-4_), in percent	1.003***	(.001)	1.002	(.001)	1.002	(.001)
Birth in 1991 (1 = yes)	1.008***	(.003)	1.007**	(.003)	1.007**	(.003)
Birth in East Germany (1 = yes)	.996*	(.002)	.998	(.002)	.991*	(.004)
Birth in 1991*East Germany	.985*	(.007)	.987	(.008)	.986	(.008)
Mother employed (1 = yes)			1.005***	(.001)	1.005***	(.001)
Non-marital birth (1 = yes)			.993***	(.001)	.993***	(.001)
Mother’s age at birth:						
15-19			1.002	(.003)	1.002	(.003)
20-24			.997*	(.002)	.997*	(.002)
25-29			Reference group		Reference group	
30-34			.999	(.002)	.999	(.001)
35-39			.995**	(.002)	.995**	(.002)
40 and older			.993	(.004)	.993*	(.004)
Mother is German (1 = yes)			1.000	(.002)	1.000	(.002)
Month of birth						
January			.999	(.003)	.999	(.003)
February			.993*	(.003)	.993*	(.003)
March			.992**	(.003)	.992**	(.003)
April			.998	(.003)	.997	(.003)
May			.997	(.003)	.997	(.003)
June			Reference group		Reference group	
July			1.000	(.003)	1.000	(.003)
August			.997	(.003)	.997	(.003)
September			.993*	(.003)	.993*	(.003)
October			.996	(.003)	.996	(.003)
November			.995	(.003)	.995	(.003)
December			.997	(.003)	.997	(.003)
Fixed effects: German states	-		Not included		Included	
N	13 863 433		13 863 202		13 863 202	
AIC	19 200 000		19 200 000		19 200 000	

Data sources: German Birth Register 1991–2010 [54] and Federal Employment Agency; East and West Germany (Berlin excluded), own calculations; unemployment data for 1990 partly based on secondary sources and imputations; ***p < .001, **p < .01, *p < .05.

However, there are also other years after 1990 in which East Germany had a very low SRB, although no major macro stressor occurred in the respective years (e.g., 2005 - see Figure 1, Panel A). This motivated us to run a sensitivity analysis to look whether replacing the 1991 dummy with a dummy for 2005 would also return a significant outcome in a model which otherwise contains the same control variables. The interaction effect between the indicator for a birth in East Germany and the one for a birth in 2005 is very similar to the respective one in Model 1 for 1991: The odds for a male birth in East Germany in 2005 are lower than those in West Germany during the same year (OR: .988; SE: .007). Also the effect of unemployment change is similar to the one in Model 1 (OR: 1.003; SE: .001)^e^.

Moving from Model 1 to Model 2, we added additional controls for maternal characteristics (employment status, marital status, nationality, and age) and for SRB seasonality (month of birth) (see Table 3). This reduced the effect of unemployment change to an odds ratio of about 1.002 (SE: .001) and that of the interaction effect indicating a birth in East Germany in 1991 to one of about .987 (SE: .008), leaving both effects not statistically significant. The size and statistical significance of these effects remain robust to the inclusion of state-level fixed effects to account for unobserved heterogeneity at the federal state level (Model 3). In order to test whether unemployment change had a stronger effect on sex determination in East Germany in 1991 than in West Germany and in later years, we included interaction effects between unemployment change at lag four and indicators for birth region (East vs. West Germany) and year (not reported here). Neither the respective two-way interactions nor the three-way interaction turned out to be statistically significant.

Among the control variables, a clear seasonal effect could be detected, with the SRB being highest in summer and winter, and lowest in early spring and autumn. This finding roughly confirms earlier results that showed the existence of seasonal variation in the SRB in Germany [55]. Among maternal characteristics, nationality seems to have no, and mother’s age at birth a non-linear effect on sex determination. Mother’s employment increases the odds of a male birth by a factor of 1.005 (SE: .001), whereas a non-marital birth is associated with a .933 lower odds for a male birth as compared to a marital birth (SE: .001). Both of these effects are statistically significant. To test if maternal employment moderates the effects of unemployment change on sex determination, we included an interaction effect between maternal employment and unemployment change at lag four. The interaction effect turned out to be not statistically significant.

## Discussion

At the opening of this paper, we emphasized the importance of the case of German reunification for evaluating the ESH with regard to sex composition at birth in human populations: As there are a large number of potential influences on sex composition at birth that cannot be empirically controlled for, the case of German reunification and the economic deterioration that selectively hit East Germany in 1991 constitutes a historical quasi-experiment that allows us to evaluate the strength of the effect of economic stress on the SRB. This case was first examined by Catalano [44]. In response to recently stated statistical challenges to research on sex ratio biases [34, 45], we set out to test the validity of the ESH against the alternative RVH, using a longer time series and more detailed data from the German birth register.

Catalano [44] argued that according to the ESH, the lowest SRB should have been recorded in 1991 when unemployment in East Germany had risen most sharply. The first part of our results, based on aggregate annual SRB data for East and West Germany from 1946–2011, confirms a drop in the East German SRB in 1991. Yet, additional deviations from the time trend in East Germany occurred in other years than 1991 which were not associated with any major increases in unemployment or other potential shocks. In 1992, for example, when the unemployment rate in East Germany continued to rise, but less sharply than in 1991, the SRB was back to its trend value. In addition, West Germany experienced considerable economic contraction due to two major oil-price shocks in the mid-1970s and early 1980s. Yet, this did not lead to clear decreases of the SRB (see Panel B, Figure 1). Therefore, we question the purported link between the SRB and economic development. In fact, in the years when unemployment rose most sharply, that is between 1973–75 and 1980–83, the SRB stayed constant or even increased. This is contrary to what the ESH would predict. In addition, downward deviations in the East German SRB in 1998, 2003, and 2005 were not associated with major increases in the unemployment rate comparable to those in the West in the 1970s and the early 1980s, or those in the East in the early 1990s.In addition to these inconsistencies in the SRB response to economic development, our aggregate data analysis also showed that even the 1991 deviation from the SRB time trend in East Germany was not statistically significant in a model that takes into account temporal autocorrelation in our time series data. This is in contrast to Catalano’s finding of a statistically significant deviation in 1991. The difference likely stems from the fact that Catalano’s analysis from 2003 covered data up to 1999, whereas we were able to include data up to 2011. This allowed us to study a longer period after reunification in 1990, which caused a drastic reduction in birth counts. The latter stayed at these low levels until the end of our observation period (see Panel D, Figure 1) and contributed to a higher volatility of the SRB in East Germany over time. We therefore suggested that this higher volatility might have made a stronger deviation in any given year more likely (RVH) and might have contributed to the low East German SRB in 1991.

Thus, for the case of German reunification, our aggregate-level analysis provided evidence against the ESH but in favor of the RVH. However, in previous work diametrical effects of stress exposure on sex determination were outlined, depending on the exact timing of stress exposure. This left the possibility open that these opposite forces might neutralize each other in annual aggregate data. We thus performed an individual-level analysis that allowed to control for monthly variation in unemployment and regional variation in the onset and intensity of the crisis. In a sensitivity analysis of various time lags of unemployment change we could show that unemployment change appeared to be associated most strongly with the sex of the child born when measured roughly in the second trimester of a pregnancy, albeit in a direction opposite of what would have been expected on the basis of the ESH. Unemployment change was not associated with the SRB when measured at the estimated time of conception. In addition, we showed that even sex ratio deviations unrelated to economic development can be statistically significant. We illustrated this for the year 2005, a year with a statistically significant downward deviation from the SRB time trend, yet with no occurrence of a major economic downturn. Thus, statistically significant deviations of the sex ratio do not per se provide support for the ESH. And the specific deviation in 1991 could as well just have occurred due to random variation. Furthermore, the effect of unemployment change, as well as the occurrence of a birth in 1991, turned out not to be statistically significant once further individual-level covariates were introduced. In addition, by testing various models with interaction effects, we could exclude the possibility that unemployment change had a stronger effect on sex determination in East Germany in 1991 than in West Germany and in later years. Thus, also our individual-level analysis, based on close to 14 million births between 1991 and 2010, showed that the drastic unemployment increases as part of the reunification process did not affect sex determination in a statistically significant way, even if we control in detail for spatial and temporal variation in unemployment change.

However, the 1991 deviation along with deviations in other years could also stem from other major macro-level stressors unrelated to economic development. The literature lists a number of potential macro events that are associated with the SRB, namely wars and natural disasters [16, 60]. While no wars occurred in Germany after World War II, there were a number of natural disasters after 1991. The three largest ones in the two parts of post-reunification Germany, measured in terms of the number of people immediately affected, were three big floods: According to data from the International Disaster Database from the Centre for Research on the Epidemiology of Disasters, one of these floods affected^f^ an estimated 330’000 individuals in East Germany and Bavaria in 2002, while the second and third biggest floods affected about 100’000 people each. Of the latter two floods one occurred in some West German regions at the turn of 1993–1994, and the other one in West German Bavaria in 1999 [61]. Although the data show SRB decreases around the years of these floods, associations with the respective flood events are not completely evident: The SRB decrease from 2002 to 2003, for instance, had already started in 2001, that is, before the 2002 flood. Two other major floods that affected parts of East Germany specifically happened in 1997 and 2006. And although SRB deviations occurred in these periods, it is unlikely that the two floods accounted for these deviations. In both cases, only a relatively small number of individuals, that is, an estimated 1000–5000 persons, were immediately affected according to the source cited above. Overall, this leaves the RVH as the most plausible explanation for the peaks and troughs in the East German SRB time series.

Also the association of maternal characteristics with sex determination needs to be discussed: Among the maternal characteristics, both whether the mother was employed or not and whether the birth was non-marital or not can be seen as indicators of individual stress. We found that the former is associated with higher odds for a male birth and the latter with lower odds for a male birth. Thus, if the assumption that maternal employment is an indicator of lower economic stress is correct, then this finding would be consistent with the economic stress hypothesis. Yet including or excluding the effect of maternal employment from our models did not significantly change the effect of state unemployment rates and the effect of a birth in 1991. Thus, even though it is consistent with the ESH applied to the individual level, this effect cannot account for the SRB deviation in 1991 or for deviations in any other year. Moreover, the lack of information on paternal employment status, part-time vs. full-time employment status, and a more fine-grained version of the parental couple’s socioeconomic status makes it difficult to provide an exact interpretation of the employment effect. This is because, theoretically, maternal employment could indicate either higher exposure to stress due to economic hardship or reduced stress due to a more secure economic position. Only information of the overall socioeconomic position of the household could resolve which of these two options is more likely in an individual case. An additional complication is that employed mothers could also experience more stress than mothers without employment in regions with high increases in unemployment rates due to fear of job loss. In order to examine whether maternal employment moderates the effect of unemployment change on sex determination, we introduced an interaction effect between maternal employment and unemployment change at lag four in a model not shown here. This interaction effect was not statistically significant, a result that speaks against the moderating role of maternal employment.

Previous research suggests that there are different effects of maternal employment on sex composition that cannot be tested with our data. For example, the Trivers-Willard effect predicts a higher share of male births among women of higher status [57, 62]. If maternal employment status corresponds to higher status, our finding is consistent with this hypothesis. However, maternal employment could also indicate low status if it is primarily a buffer against unemployment of the male partner. Empirical research on maternal employment has shown that having a highly stressful job can be associated with an increased probability for a female birth [4]. In this case, our finding would be inconsistent with these results. Thus, future research should try to test the economic stress hypothesis at the individual level with a more complete measurement of both parents’ employment and socioeconomic status. As new administrative data for research purposes is increasingly being made available, there is reason to hope that we will have access to appropriate data for conducting such a test in the medium term.

## Conclusion

Drawing on new and richer data, and using more sophisticated statistical methods to deal with changing volatility in the SRB over time, our re-analysis of the German reunification case to look at the effects of economic contraction on SRB provides little support for the economic stress hypothesis (ESH). We rather find support for our random variation hypothesis (RVH) which postulates that the low SRB in East Germany in 1991 stems from higher annual random fluctuations of the SRB as a result of a substantial reduction in birth counts after 1990. Against the background of other findings that do show associations between other types of stressors and even economic stress with SRB fluctuations [10, 11, 17], it appears surprising that in this particular case no such association can be found. One observation that may explain this inconsistency could be related to particularities of the German case: even though East Germany underwent a rapid increase in unemployment rates in 1991 and continued to experience high unemployment rates in the years that followed, many of the adverse consequences were buffered by a generous welfare state. Therefore, the effects of economic stress on the SRB may also have been less severe.

Another explanation for not finding a significant effect might be that the stress induced by the economic and political transformation in East Germany after 1991 rather affected fertility decisions than the SRB: Transformation in East Germany and in other East and Central European countries led women to postpone fertility [63]. In East Germany, fertility plummeted right after reunification and partial recuperation of fertility levels took several years [64]. Such behavioral adjustments may reduce physiological effects of economic stress on the SRB, particularly if behavioral adjustment is strongest among individuals who are affected most severely by an economic downturn. Considering such behavioral adjustment processes in future research would help reconcile previously mixed findings on the association of stressors on the macro-level and SRB fluctuations. This is illustrated when comparing the terrorist attacks of Sept. 11, 2001 and their SRB decreasing effect [10, 11] with the case of German reunification and the null-effect on the SRB: In the former case the attack occurred suddenly and women did not have time for behavioral adjustments. Thus, more pregnancies were at risk of spontaneous abortion due to the stresses associated with the attacks. In the latter case, however, several months passed between the fall of the Berlin Wall in 1989 and the start of the drastic increase in unemployment rates in East Germany in the middle of 1990. Thus, the perceived insecurities of the transformation process in 1989 and 1990 may have prompted a large share of women to postpone their childbearing plans to the future. This in turn reduced the number of pregnancies that would otherwise have occurred and been at risk of spontaneous abortion during the period of highest economic insecurity and stress. Future research on the effects of stress on the SRB should therefore take into account both behavioral and physiological adjustments to stress.

## Endnotes

^a^For 14 of the 16 German states, the birth register covers all years from 1991–2010. We lack the years 1991–1994 for the East German state of Mecklenburg-Vorpommern, and the year 1991 for the West German state of Saarland. The birth register data unfortunately do not allow us to distinguish between West and East Berlin which belonged to West and East Germany, respectively, before reunification.

^b^See Endnote ^a^.

^c^For inclusion in the birth register, recording paternal age is mandatory for marital births only. While no information on the father was collected if the birth was non-marital until 1999, since 2000 the parents of non-marital children can choose whether they want the paternal age to be reported. Given that in East Germany in the 1990s and the first decade of the 2000s 40%-60% of the births were non-marital, we lack information on the paternal age for a substantial share of the births.

^d^As an artifact of the way the moving average is calculated, that is, as the average variance across the focal year and the four subsequent years, this rise slightly predates reunification.

^e^The odds ratios for the main effects of birth year (2005) and birth region (East Germany) were .990 (SE: .003; p: .000) and .996 (SE: .002; p: .018), respectively.

^f^According to the source, the number of people affected includes people injured, left homeless, and those "requiring immediate assistance during a period of emergency; it can also include displaced or evacuated people."

## Statistical Appendix

Every model in Table 1 (Model 1–5) includes a constant and the following variables: year of measurement (t, with a range from 1946 to 2011), the West German SRB in a given year t (SRB^West^), and a dummy variable indicating whether the year is 1991 or not (DUMMY91). Thus the following part is fixed in each model:
1

Model 1 is an ordinary least squares regression:
23

Model 2 is an ARMA (1,1) time series regression. This indicates that we included an autoregressive component (AR) and a moving average (MA), both at a time lag of one year.
45

Model 3 is an ARMA(1,1), ARCH(2) model where ARCH stands for "autoregressive conditional heteroskedasticity" and the number stands for a time lag of two years. That is, as opposed to Models 1 and 2, here errors are not assumed to have constant variance σ^2^. Instead, the variance of the errors (h_t_) is allowed to change over time depending on the volatility of errors in the previous two years (lag two).
678

In Model 3, to obtain the error variance h_t_, the parameters ω, α_1_, and α_2_ are to be estimated based on previous errors with a time lag of two years. For the estimation we used the garchFit function in the R add-on package fGarch. The function did not allow to estimate the regression and variance parameters in one step. We thus used a three-step procedure as recommended in the statistical literature [52]. First, we obtained the regression parameters using ordinary least squares (Model 1). Second, we estimated the variance parameters ω, α1, and α2 from the raw residuals from Model 1. Finally, we re-estimated the regression parameters using a weighted least squares regression with the reciprocal of h_t_ as weight:
9

Models 4 and 5 are replicates of Model 1, but with weighted least squares to adjust for heteroskedasticity in the data. In Model 4, weights are based on the number of births in East Germany in a particular year (n_t_^East^), divided by the total number of births in East Germany between 1946 and 2011 (N^East^).
10

For Model 5 we multiplied the two weights used in Models 3 and 4 [53]:
11

For each of the Models 1–3 we tested a series of alternative models. Model 1 had the best fit in a series of six models in which we tested several specifications by in- or excluding the following variables: year of measurement, year of measurement squared, and the West German SRB time series. Model 2 had the best fit in a series of models with moving averages and autoregressive components including different time lags. Model 3 had the best fit in a series of four different ARCH and generalized ARCH models, applying different time lags. The ARCH(2), or GARCH(2,0), model turned out to be the best one according to the AIC value.

## Abbreviations

ARCH: Autoregressive conditional heteroskedasticity
ARMA: Autoregressive moving average
ESH: Economic stress hypothesis
RVH: Random variation hypothesis
SRB: Sex ratio at birth.
